# Synergistic and antagonistic effects of mixing monospecific soils on plant-soil feedbacks

**DOI:** 10.1007/s11104-018-3694-6

**Published:** 2018-05-31

**Authors:** Hai-kun Ma, Ana Pineda, Andre W. G. van der Wurff, T. Martijn Bezemer

**Affiliations:** 1 Department of Terrestrial Ecology, Netherlands Institute of Ecology (NIOO-KNAW), P.O. Box 50, 6700 AB Wageningen, The Netherlands; 2 Institute of Biology, Section Plant Ecology and Phytochemistry, Leiden University, P.O. Box 9505, 2300 RA Leiden, The Netherlands; 3 Delft Research Group, Section Green Projects, Groen Agro Control, P.O. Box 549, 2600 AM Delft, The Netherlands

**Keywords:** Plant-soil feedback, Plant health, Additivity, Interaction, Species-specific soil

## Abstract

**Background and aims:**

Plants influence the soil they grow in, and this can alter the performance of other, later growing plants in the same soil. This is called plant-soil feedback and is usually tested with monospecific soils, i.e. soils that are conditioned by one plant species. Here, we test if plant-soil feedbacks of inocula consisting of mixtures of monospecific soils can be predicted from the effects of the component inocula.

**Methods:**

Chrysanthemum plants were grown in sterile soil inoculated with eight monospecific conditioned soils and with mixtures consisting of all pairwise combinations. Plant biomass and leaf yellowness were measured and the additivity was calculated.

**Results:**

On average, plant biomass in the mixed inocula was slightly but significantly (6%) lower than predicted. In contrast, when growing in mixed inocula, plants showed 38% less disease symptoms than predicted. Moreover, the larger the difference between the effects of the two monospecific soils on plant growth, the higher the observed effect in the mixture exceeded the predicted effects.

**Conclusions:**

We show that mixed monospecific soils interact antagonistically in terms of plant growth, but synergistically for disease symptoms. Our study further advances our understanding of plant-soil feedbacks, and suggests that mixing soils can be a powerful tool to steer soil microbiomes to improve plant-soil feedback effects.

**Electronic supplementary material:**

The online version of this article (10.1007/s11104-018-3694-6) contains supplementary material, which is available to authorized users.

## Introduction

Plants are an important determinant of the composition of soil communities, and the effect of a plant on the soil microbial community can subsequently affect the performance of other plants that grow later in that soil, a phenomenon termed plant-soil feedback (van der Putten et al. 2013; Bever et al. 1997). Such plant-soil feedback effects are typically recorded as the net outcome of all negative and positive effects on plant growth. However, a single plant can increase the density of soil organisms with both negative (e.g. soil pathogens) and positive (e.g. beneficial soil organisms such as plant growth promoting bacteria) effects (Mendes et al. 2013; Raaijmakers et al. 2009). An important question that has received little attention is how mixing soils conditioned by different plant species, each with positive and negative effects, influences the net effect of this soil on plant performance.

When mixing soils or in fact any two characteristics, three possible effects can be expected: synergistic, additive, or antagonistic. First, the outcome of mixing two specific soil communities can be stronger than the two individual effects together (synergistic effect). For example, Hendriks et al. (2013) found that when the same amount of soil was added, mixtures of soil collected from different monocultures sustained higher plant biomass than pure monoculture soils. On the contrary, mixing soil communities could also lead to antagonistic effects, so that the mixed effects are weaker than what would be predicted from the individual effects. Several studies reported, for example, that combinations of biocontrol microbial strains fail to reduce specific plant diseases, even though the individual strains all have suppressing effects on the disease, suggesting that antagonistic interactions occur among these microbial strains (Schisler et al. 1997; Sarma et al. 2015). Third, it is also possible that positive and negative interactions between plants and soil organisms counterbalance each other, so that the mixed soil effect is simply the sum of individual effects (additive effects; Singh et al. 2015). Ladygina et al. (2010), for example, showed that when added in isolation, arbuscular mycorrhizal fungi increased plant community productivity, while addition of soil decomposers decreased productivity, and addition of root herbivores had no effect. When these three groups of soil organisms were added together, their effect on productivity could be predicted from adding up the individual negative and positive effects. Due to the potential for interactions between soil microorganisms, whether plant performance in mixed soil communities can be predicted from the plant performance in the soils conditioned by a single plant species (i.e. monospecific soils), is an open question.

Whether plant-soil feedback effects in mixtures of monospecific soils, are additive, synergistic or antagonistic may depend on how different the effects of the monospecific soils are, but to our knowledge, there are no studies yet that have tested how the difference between two monospecific soils influences the effects on plant growth or plant health. However, from plant competition experiments it is known that synergistic effects occur more often when characteristics of the two species that compete differ considerably. Growing together two species that occupy different niches, allows the species to capture resources in ways that are complementary, leading to aboveground overyielding (Mommer et al. 2010; Cardinale et al. 2007). Similarly, decomposition experiments have shown that mixing plant species-specific litters that differ greatly in chemistry leads to higher than expected decomposition rates, but this is not true when the different litters are relatively similar in chemical composition (Harguindeguy et al. 2008). Thus, when mixing two factors (e.g. two plant species-specific litters) that greatly differ in composition or effect, the net effect of the mixture tends to be better than predicted. Hence, we may also expect that mixing two monospecific soils with distinctly different soil communities, and thus with largely different effects on plant growth should result in a more positive effect of plant growth than what is predicted based on the sum of the effects of the individual soil communities.

In this study we examine how mixing soils conditioned by different plant species influences net plant-soil feedback effects on plant growth and leaf yellowness (a plant health indicator) (Reddy 2016). In a previous study, we tested the plant-soil feedback effects of 37 different plant species and observed that inoculation of soil conditioned by several species led to increased growth and resistance against *Pythium*, while inoculation of soils conditioned by other species reduced growth and resistance (Ma et al. 2017). In the current study, we selected eight plant species (that previously showed positive and negative soil effects on chrysanthemum growth) and examined the effects of mixing these plant species-specific soil inocula on chrysanthemum performance. Specifically, we ask: (i) can the effects of mixed soil inocula be predicted from the effects observed with the monospecific soil inocula that are used for the mixture? (ii) is such effect synergistic, additive, or antagonistic? and, (iii) how is this related to the absolute difference between the effect of the two monospecific inocula? For each inoculum we also examined how its effect is influenced by mixing it with other inocula.

## Materials and methods

### Plant material

The focal plant in our study is *Dendranthema X grandiflora* (Ramat.) Kitam. cv. Grand Pink (Chrysanthemum, syn. *Chrysanthemum* X *morifolium* (Ramat.) Hemsl., Asteraceae). Chrysanthemum is one of the major cut flower crops that is cultivated in soil in glasshouses. The soil is sterilized regularly by steaming to control soil pathogens (Thuerig et al. 2009; Tamm et al. 2010). Hence, in this system the use of inoculating conditioned soil inocula into sterilized bulk soil represents a realistic scenario. Chrysanthemum cuttings were provided by the breeding company FIDES by Dümmen Orange (De Lier, The Netherlands).

### Experimental set-up

The experiment consisted of two phases, in the first phase, the conditioning phase, we grew eight plant species in monocultures to create monospecific soils. In the second phase, the test phase, we used mixtures of all combinations of two monospecific soils (including mixtures of two identical monospecific soils), and used these soils as inocula to test the effects on chrysanthemum growth.

#### Phase I: Conditioning phase

For the conditioning phase, soil was collected (5–20 cm deep) in June 2015 from a former arable field, which has become a natural grassland since 1996 (Mossel, Ede, The Netherlands). The sandy-loam soil was homogenized and sieved (1 cm mesh size) to remove coarse fragments and all macro-arthropods. Pots (13 × 13 × 13 cm) were filled with a homogenized mixture of field soil and sterilized field soil in a 1:1 ratio. The sterilized soil was added to minimize potential differences in soil nutrients and to provide a niche for the soil microbes to grow and hence increase the potential for plantspecies-specific effects on the soil community. Pots were filled with 1.6 Kg of soil (based on dry weight). Soil sterilization was done by gamma irradiation (> 25 K Gray gamma irradiation, Isotron, Ede, The Netherlands).

Eight plant species were used to condition the soils: *Anthoxanthum odoratum* (AO), *Bromus hordeaceus* (BH), *Festuca filiformis* (FF), *Lolium perenne* (LP), *Holcus lanatus* (HL), *Rumex acetosella* (RA), *Galium verum* (GV) and *Hypochaeris radicata* (HR). Seeds of all species were obtained from a wild plant seed supplier (Cruydt-Hoeck, Assen, The Netherlands). Seeds were surface sterilized in 3% sodium hypochlorite solution for 1 min, rinsed and germinated on sterile glass beads in a climate chamber at 20 °C (16 h/8 h, light/dark).

Five one-week-old seedlings were transplanted in monocultures in each pot, and there were ten replicate pots for each species. In total, the conditioning phase comprised of 80 pots (monocultures of 8 plant species × 10 replicates). Seedlings that died during the first week of the experiment were replaced. As a few seedlings died later, after two weeks, the number of seedlings in each pot was reduced to four so that the density was the same in all pots. All pots were placed randomly in a greenhouse with 70% RH, 16 h 21° (day) and 8 h 16° (night). Natural daylight was supplemented by 400 W metal halide lamps (225 μmol s^−1^ m^−2^ photosynthetically active radiation, one lamp per 1.5 m^2^). The pots were watered by hand every other day. Ten weeks after transplanting, the plants were carefully removed from each pot and the largest roots were removed from the soil as they may act as a source for re-growing plants. Finer roots were left in the soil as the rhizosphere around these roots may include a major part of the microbial rhizosphere community. The soil from each pot was homogenized and stored separately in a plastic bag at 4 °C until used in the test phase so that there were 10 replicate soils for each plant species. The soils are called “soil inocula” hereafter.

#### Phase II: Test phase

For the test phase, the conditioned soil from the first phase was used as inoculum. There were two types of inocula, monospecific inocula (i.e. soil conditioned by one plant species), and heterospecific soil inocula (i.e. 1:1 mixtures of two monospecific conditioned soils). Mixtures of all combinations were used, thus the feedback phase comprised of 360 pots (28 combinations of mixed inocula × 10 replicates + 8 conspecific mixtures × 10 replicates). Pots of 1 L (11 × 11 × 12 cm; length × wide × height) were filled with a homogenized mixture of 10% inoculum and 90% sterile field soil (see above). Two 5 cm chrysanthemum cuttings (without roots) were planted in each pot. Prior to planting, the soil in each pot was well watered and 100 ml half-strength Hoagland nutrient solution was added (Li and Cheng 2015). The pots were randomly placed on trolleys, each trolley had 48 pots and was tightly covered with a thin transparent plastic film for 10 days to create a closed environment with high humidity that favors rooting. After 10 days, most of the cuttings had rooted. Non-rooted cuttings were removed and from pots where both cuttings had rooted, a randomly selected chrysanthemum cutting was removed. Plants were fertilized following grower practice: half-strength Hoagland nutrient solution (0.9 mS/cm electric conductivity) for the first two weeks, and full strength Hoagland solution (1.4 mS/cm electric conductivity) during the following two weeks. For the last two weeks, the strength was increased to 1.6 mS/cm electric conductivity. The density of pots on each trolley was reduced two weeks after the beginning of the second phase to 32 pots per trolley so that there was 10 cm space between each pot. All pots were randomly arranged in a greenhouse compartment kept under the same conditions as described for the conditioning phase.

### Plant performance

Eight weeks after planting the cuttings, all plants were harvested. For each plant, the number of leaves that showed yellowness and the total number of leaves were recorded. Leaf yellowness in chrysanthemum is symptomatic for diseases such as those caused by soil pathogens like *Verticillium*, *Fusarium* or *Puccinia* (Reddy 2016). The characterization of yellowness was based on observations by eye, and for all leaves which were characterized as yellow, an area of at least 5% of the leaf was yellow. Yellowness was then calculated as the proportion of yellow leaves (number of yellow leaves relative to the total number of leaves on that plant). Plants were clipped at soil level and roots were washed over a sieve (2 mm mesh). Shoot and root biomass was then oven-dried (60 °C for 3 days) and weighed. Plant biomass was calculated as the sum of plant shoot and root dry weight.

### Calculations and statistical analysis

The predicted (additive) effects of mixed inoculum (e.g. combination AB) on chrysanthemum biomass and yellowness were calculated as (effect of inoculum A + effect of inoculum B)/2. This was done for each soil replicate separately. Then, the observed effects of mixed inocula were compared with their predicted effects. If there is no significant difference between these two effects, this indicates that the effects of mixing are additive. A significantly lower than predicted effect indicates antagonistic interactions, while a significant higher effect indicates synergistic interactions. In this analysis, we used each mixture as a replicate. For this, we averaged the values of the replicate samples of each mixture. A paired t-test was used to test if the observed effects of mixing inocula (real values) were significantly different from the predicted effects. This analysis was done for chrysanthemum biomass and yellowness. For the statistical analysis, chrysanthemum yellowness was arcsine-transformed, as yellowness was entered as proportional data. The average effect for all inocula combinations is presented in the main text. The detailed results for each mixture (i.e. each combination of two monospecific soils) are presented in the supplementary materials (Fig. S1).

To examine whether there was a relationship between the difference among two monospecific inocula on chrysanthemum performance and the difference between the observed and predicted effects when mixing these two inocula, we used linear regression. We first calculated the absolute difference between the effects of the two monospecific inocula, and this was plotted against the difference between the observed and the predicted effect of the mixture. In this latter calculation, positive or negative values indicate synergistic or antagonistic interactions between component monospecific inocula respectively. Data were checked for homogeneity of variance and normality by inspection of the residuals before the analysis. We then determined the sign and strength of the linear relationship between these two parameters.

To examine for each conditioning species the effects of mixing on plant biomass and leaf yellowness, we compared the eight inocula that contained each conditioning species using a one-way ANOVA. Individual comparisons were based on a post-hoc Tukey test. The response of each monospecific inoculum to mixing was determined by comparing the effects of the heterospecific mixtures containing a monospecific inoculum to the effect of the monospecific inoculum: (response of inoculum A to mixing = the average effect of heterospecific mixtures containing inoculum A – the effect of monospecific inoculum A). This was done for each replicate separately. A one-sample t-test was used to test for each inoculum if the response was significantly different from zero. Values that are not different from zero indicate that the response is not different from the monospecific mixture, values less than zero indicate that heterospecific mixing has a negative influence, while values larger than zero indicate that mixing has a positive effect. One-way ANOVA was used to determine if these mixing effects on biomass differed between inocula, and a generalized linear model was used to analyze differences in yellowness. The chrysanthemum biomass and yellowness in each mixed inoculum are listed in Table S1 and Table S2 of the supplementary materials.

To test whether there were significant differences between mixtures which contained a specific monospecific inoculum on plant biomass and yellowness, we used one-way ANOVA. A post-hoc Tukey test was used for pairwise comparisons between different mixed inocula. All analyses were performed in R (version 3.0.1, R Development Core Team, 2017).

## Results

**Fig. 1:**
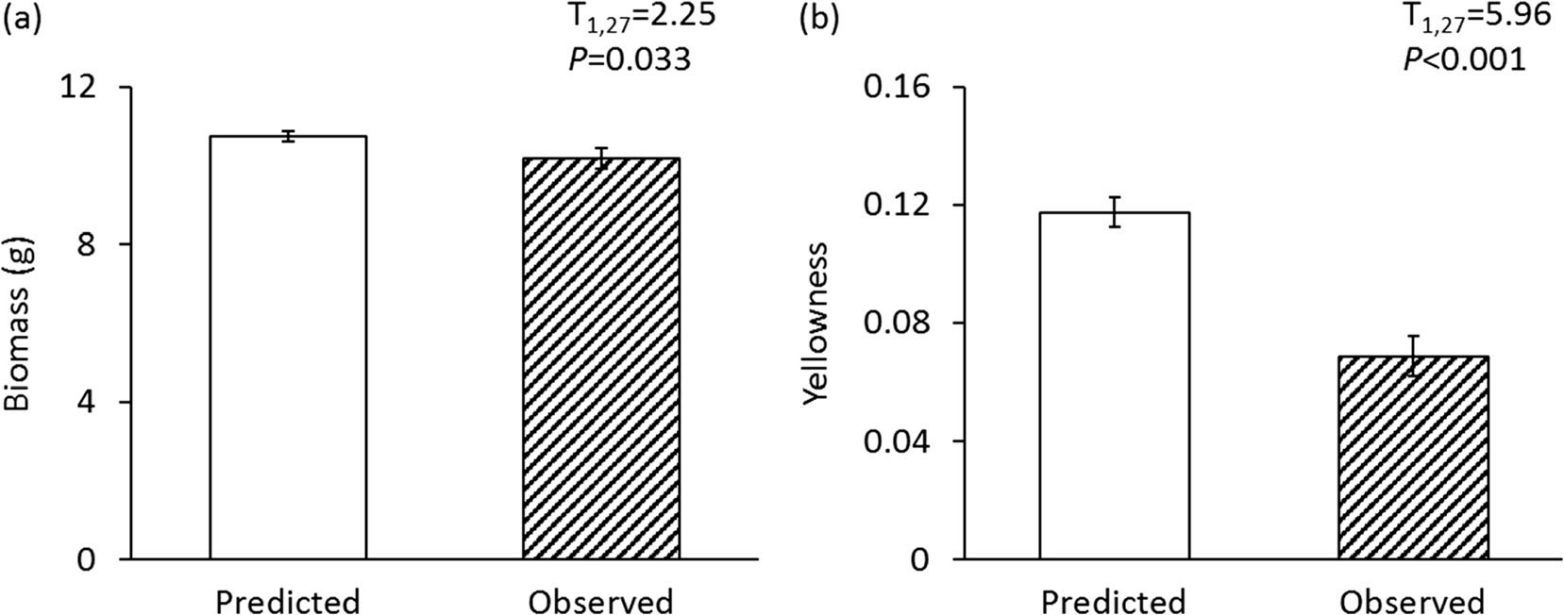
Mean (± SE) predicted (white bars) and observed (hatched bars) effects of soil mixing on chrysanthemum biomass (**a**) and yellowness (**b**). White bars represent predicted effects of mixed inocula based on effects in component monospecific inocula (effect of inoculum A + effect of inoculum B)/2. T and *P* values from a paired t-test are also presented. The figure shows the average effects of all mixtures. The effects for each separate two-species soil mixture are presented in Fig. S1

The biomass of plants exposed to mixed soil inocula was lower than what was predicted from the effects of the monospecific inocula, suggesting that on average two soil communities interact antagonistically with respect to plant growth. However, leaf yellowness was also lower than predicted and therefore soil mixing benefited plant health (Fig. 1). With regard to each monospecific inoculum, for four out of eight plant species, observed chrysanthemum biomass was significantly lower in mixtures than predicted. For two out of eight species leaf yellowness was significantly lower in mixtures than predicted (Fig. S1).

**Fig. 2:**
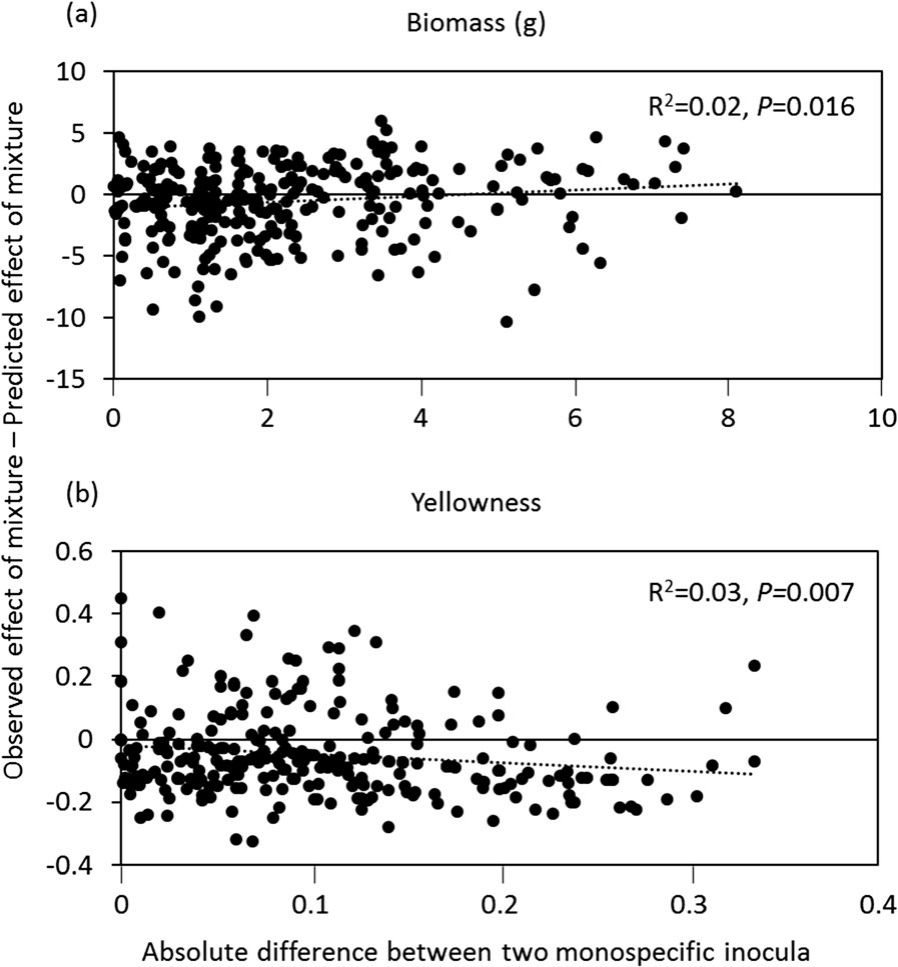
Relationship between the difference among two monospecific inocula on plant biomass (**a**) and yellowness (**b**), and the difference between the observed and predicted effects when mixing these two inocula. The difference of monoculture inocula is calculated as (|effect of inoculum A – effect of inoculum B|). The difference between observed and predicted effects of the mixtures is calculated as (observed value of mixture A + B – predicted value of mixture A + B). The goodness of fit (R^2^) and *P* value of both regressions are also presented

For total plant biomass, there was a weak but significantly positive relationship between the absolute difference among the two monospecific inocula and how much the observed effects of their mixture varied from the predicted effects (Fig. 2a). This means that the larger the difference between the effects of the two monospecific soils on plant growth is, the higher the observed effect of the mixture exceeds the predicted effect. The difference between observed and predicted yellowness became more negative with increasing differences between the effects of the two component inocula (Fig. 2b). This relationship was not driven by the inoculum with most extreme effects, *H. radicata*, since removal of this species-species soil inoculum from the analysis did not alter the trend (Fig. S2).

**Fig. 3:**
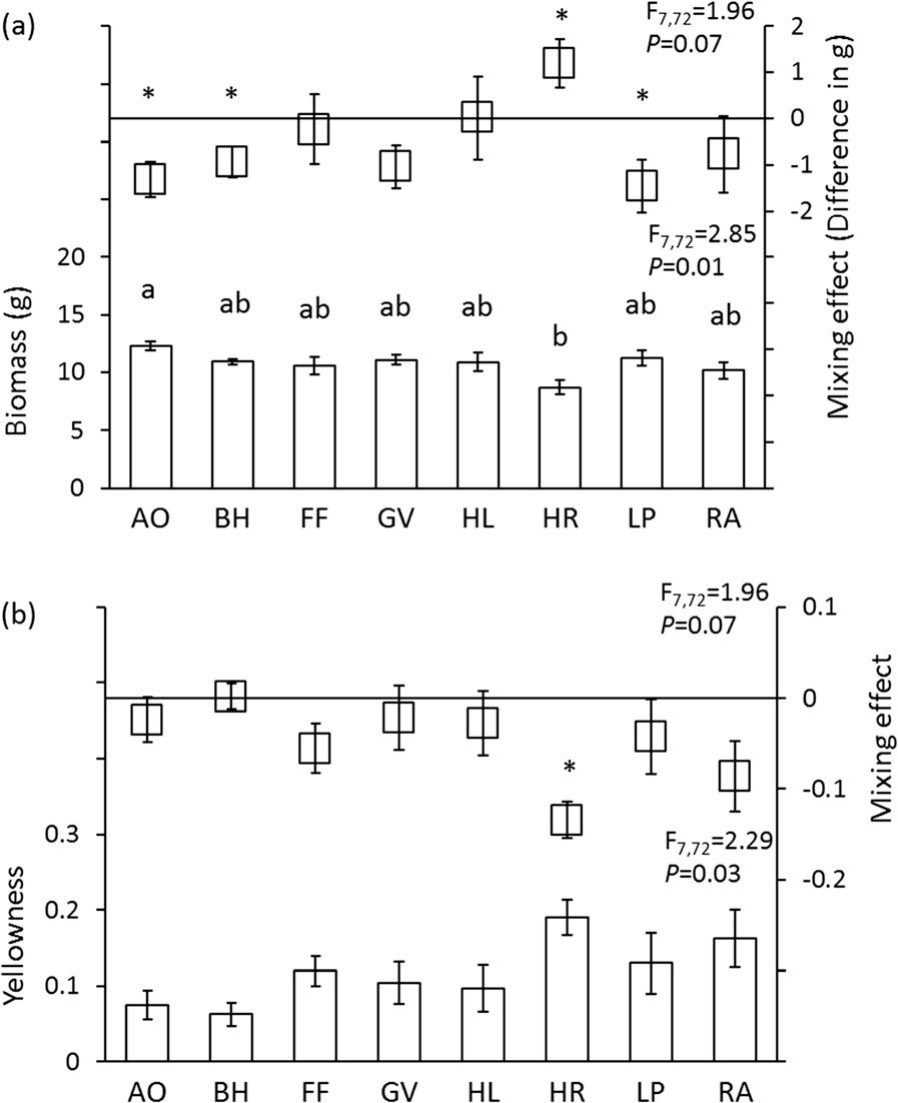
Effects of monospecific soil inocula on chrysanthemum biomass (**a**), and yellowness (**b**) in conspecific and heterospecific mixtures. Mixing effects are calculated as (average effects of heterospecific mixtures that include inoculum A – effects of monospecific inoculum A). The zero line indicates that mixing does not differ from the effects of the monoculture species inocula. * represents significantly different from zero (one-sample t-test, *P* < 0.05). The bars represent the effects of each monospecific inoculum (mean ± SE). F and *P* values from a one-way ANOVA are also presented. Bars with identical letters are not significantly different from each other based on a post hoc Tukey test. Species abbreviations are explained in the Materials and methods section

Overall, chrysanthemum biomass differed significantly among monospecific inocula. Greatest gain in chrysanthemum biomass was observed when grown with monospecific *A. odoratum* inoculum, and lowest with *H. radicata* inoculum (Fig. 3a). On average, plant biomass in heterospecific mixtures was significantly lower than in conspecific mixtures for inocula that included soil conditioned by *A. odoratum*, *B. hordeaceus* and *L. perenne*. Mixing soil conditioned by *H. radicata*, the most negative monospecific inoculum, with other inocula resulted in more biomass than when chrysanthemum was grown in monospecific soil conditioned by *H. radicata* (Fig. 3a). Leaf yellowness did not differ between monospecific inocula. Yellowness in heterospecific mixtures did not significantly differ from those in conspecific mixtures, except for soil conditioned by *H. radicata*, where heterospecific mixing resulted in lower levels of leaf yellowness (Fig. 3b).

When inocula conditioned by *A. odoratum*, *B. hordeaceus*, *F. filiformis* and *H. lanatus* were mixed with other inocula, this did not lead to differences between these mixtures on plant biomass. In contrast, mixing inocula conditioned by *R. acetosella*, *H. radicata*, *G. verum* or *L. perenne* with other inocula resulted in significant differences between these mixtures on plant biomass, as mixing of *L. perenne* with inocula conditioned by forbs resulted in lower biomass (Table S1). Leaf yellowness did not differ in these comparisons (Table S2).

## Discussion

Our study shows that plant-soil feedback effects that arise from mixing monospecific conditioned soils are on average non-additive. In this experiment, the biomass and the yellowness of plants growing in pots with mixed inocula were significantly lower than what was predicted from the effects of the monospecific inocula. Moreover, when the difference in the effects between two monospecific inocula increased, the effects on plant biomass and health when mixing these two inocula became weakly positive than expected. This suggests that synergistic interactions in soil microbial communities increase when the effects of the two monospecific inocula are more different, implying that the synergistic or antagonistic effects of soils on plant growth can be predicted based on the difference between their individual effects.

Plant biomass was not enhanced by mixing plant monospecific soils, but leaf yellowness was reduced. The observed reduction in plant biomass and leaf yellowness relative to the effects predicted from the monospecific soils could be due to several reasons. First, the mixed inocula consisted of 50% of both monospecific soils, and as such only consisted of 50% of the density of soil microorganisms of both monospecific soils. Previous studies found that a reduction in volume of a soil inoculum reduces the effect of the inoculum on the plant (St-Denis et al. 2017; Mendes et al. 2011; Hol et al. 2017). However, whether the relative reduction of the effectiveness of the soil inoculum is linearly or non-linearly related to the change in soil volume is unknown. Our results suggest that when the volume of one monospecific soil in the inoculum is reduced by 50%, the effects of the soil microorganisms on plant growth are reduced by more than 50%, as the mixed inocula had weaker effects than what was predicted. Thus, the observed reduction in plant biomass and leaf yellowness may be due to the weakened effects of beneficial or detrimental microbes in mixed inocula. Second, mixed soils most likely harbor a higher microbial diversity than monoculture soils, and this may increase the likelihood of introducing in the mixture both detrimental and beneficial organisms that will interact with the plant. However, the observed chrysanthemum leaf yellowness which is presumably caused by soil pathogens was also reduced, and this indicates that soil pathogens are not the reason of the reduction in plant biomass in mixed inocula. Instead, it is possible that enhanced plant health may be at the cost of plant growth as interacting with beneficial soil microbes can be costly for plants (Morgan et al. 2005). However, such interaction can also provide extra functions such as disease suppression or induced resistance (Pieterse et al. 2014; Mendes et al. 2011), as we observed in terms of leaf yellowness. It is important to note that, in this study, we only recorded plant performance during one growth cycle and that the soil-mediated effects reported here may become stronger during subsequent plant growth cycles when the soil community has developed further.

The fact that mixing monospecific soils leads to non-additive effects on plant growth is in line with other studies that reported non-additive effects of mixing soils from different origins on plant growth (Brandt et al. 2013; Burns et al. 2017). Brandt et al. (2013) found that plants grew worse in homogenized mixtures of soils that are of different origins than what would be predicted from the effects observed in plants grown in monospecific soil. Later, Burns et al. (2017) showed that the composition of soil microbial communities in soil mixtures differs from that in monoculture soils. They proposed that the influence of the microbial community on plants could be either via direct effects of soil microbes on the plant or via indirect effects of soil microbes on soil nutrient availability for the plant. In their study, the pots contained 100% live soil. In contrast, in our study we inoculated 90% sterile soil with 10% live conditioned soil to homogenize abiotic conditions (Kardol et al. 2006). Furthermore, in our experiment, all soils received high levels of fertilization, following farmer’s practices further minimizing differences in abiotic conditions (i.e. nutrient levels). Therefore we suggest that the mixing effects that we observed on plant performance were likely to be caused by interactions between soil microbes (Brinkman et al. 2010). In the studies by Brandt et al. (2013) and Burns et al. (2017), the effects in homogenized soil mixtures were compared with effects in heterogeneous monospecific soil, and differences in patchiness between different soil treatments may have an important impact on the results (Wubs and Bezemer 2016). In our study, we compared the homogenized soil mixtures with homogenized monospecific soils, thus narrowing down the number of factors that could potentially influence the results. To our knowledge, no study has tested both responses in terms of plant growth and plant health to soil mixing. The leaf yellowness results in our study provide important information about the negative influence that certain soils can have on plant health, and how such negative effects can be reduced by mixing soils.

Our results show that there was a weak relationship between the magnitude of the difference between the effects of two monospecific soil inocula on plant growth and how much the observed effect differed from the predicted effect. This trend did not change when we excluded mixtures that contained soil conditioned by *Hypochaeris radicata* from the analysis, the soil inoculum that had the most negative influence on chrysanthemum performance. This result has three implications, first, when mixing two monospecific soils with similar positive effects on plant growth, the effect of the mixture will be worse than the sum of their individual effects. Similarly, mixing two monospecific soils with similar negative effects will not reduce the negative effect more than what would predicted from the monospecific soil effects. Third, when mixing two soils that have opposing effects, the effect of the mixture tends to be more positive than the sum of their individual effects.

The effects discussed above are on plant growth, with regard to yellowness, there were only additive to synergistic interactions (in terms of plant benefits). Our results therefore suggest that mixing two inocula will alleviate negative effects of monospecific inocula on plant health. Mixing two soils with different microbial communities (and we expect with large differences in their effect on plant growth) can lead to synergistic effects if adding a second soil will be complementary to the existing microbial community. This is in accordance with studies about mixing plant species or plant litters, which have found that synergistic interactions are likely to happen when the two species or litters have very different characteristics (Mommer et al. 2010; Cardinale et al. 2007; Harguindeguy et al. 2008; Gartner and Cardon 2004). Further studies should examine the differences in soil microbial composition before and after mixing.

In summary, this study demonstrates that the plant-soil feedback effects of monospecific conditioned soils are non-additive when mixed. On average, plants show less disease symptoms but also grow worse in soil with mixed inocula compared with prediction. Moreover, with increasing differences among the effects of two soil inocula on plant growth and health, the synergistic effects also increase when the soils are mixed. The synergistic and antagonistic effects of soils are two extreme outcomes in the wide range of potential interactions that can occur. We created an antagonistic to synergistic continuum and such continuum could provide important information about predicting the effect of mixing two soils on plant. For example, if our results can be extended to other systems, we may be able to select soil inocula that vary greatly in how they affect plant growth and mix them, in order to create synergistic interactions. This study therefore exemplifies how soil microbiomes can be manipulated to enhance disease resistance (Pineda et al. 2017). Our study with the cut flower chrysanthemum also highlights the role and potential of using plant-soil feedbacks in influencing the health and yield of a horticultural crops (Dias et al. 2015; Pineda et al. 2017).

## Electronic supplementary material

ESM 1 (DOCX 115 kb)
