# Supplementary material for: Synergistic and antagonistic effects of mixing monospecific soils on plant-soil feedbacks
Source: Plant Soil. 2018 May 31;429(1):271–9. doi: 10.1007/s11104-018-3694-6 (PMC6434923; doi:10.1007/s11104-018-3694-6)

Supplementary Material

Synergistic and antagonistic effects of mixing monospecific soils on plant-soil feedback

**Hai-kun Ma*, Ana Pineda, Andre W.G. van der Wurff, T. Martijn Bezemer**

*** Correspondence:** Corresponding Author: [H.Ma@nioo.knaw.nl](mailto:H.Ma@nioo.knaw.nl)

**Table S1** Plant biomass in each mixed inoculum (mean + SE). Each row/column combination indicates the average biomass for this inocula combination. For each column, the F value from a one-way ANOVA is also presented. *,**,*** indicate significant differences at P<0.05, P<0.01 or P<0.001, respectively. Within each column, values followed by identical letters are not significantly different based on a post hoc Tukey test. The shaded part of the table is presented twice. Plant species abbreviations are described in the material and methods section.

| Inocula | AO | BH | FF | GV | HL | HR | LP | RA |
| --- | --- | --- | --- | --- | --- | --- | --- | --- |
| AO | 12.3+0.4a | 10.1+0.8a | 11.0+1.2a | 10.9+ 0.5b | 10.1+0.8a | 10.5+ 0.7ab | 12.9+0.3c | 11.6+ 0.8b |
| BH | 10.1+0.8a | 10.9+0.3a | 8.8+1.0a | 10.5+ 1.2ab | 10.3+0.9a | 9.6+ 0.8ab | 11.4+0.7c | 9.2+ 0.5ab |
| FF | 11.0+1.2a | 8.8+1.0a | 10.6+0.8a | 10.7+ 0.6ab | 12.7+0.8a | 9.7+ 0.9ab | 10.4+0.6bc | 9.1+ 0.9ab |
| GV | 10.9+0.5a | 10.5+1.2a | 10.7+0.6a | 11.1+ 0.4b | 10.4+0.6a | 11.2+ 0.8b | 7.5+1.1ab | 9.1+ 0.4ab |
| HL | 10.1+0.8a | 10.3+0.9a | 12.7+0.8a | 10.4+ 0.6ab | 10.9+0.8a | 10.9+ 0.5ab | 11.7+0.8c | 10.1+ 1.2ab |
| HR | 10.5+0.7a | 9.6+0.8a | 9.7+0.9a | 11.2+ 0.8b | 10.9+0.5a | 8.7+ 0.6ab | 7.7+0.5ab | 9.7+ 1.1ab |
| LP | 12.9+0.3a | 11.4+0.7a | 10.4+0.6a | 7.5+ 1.1a | 11.7+0.8a | 7.7+ 0.5a | 11.3+0.7c | 7.1+ 0.9a |
| RA | 11.6+0.8a | 9.2+0.5a | 9.1+0.9a | 9.1+ 0.4ab | 10.1+1.2a | 9.7+ 1.1ab | 7.1+0.9a | 10.2+ 0.7ab |
|  |  |  |  |  |  |  |  |  |
| Mixture(df=7,72) | 1.88 | 1.16 | 1.90 | 2.99** | 1.11 | 2.26* | 9.17*** | 2.23* |

| Inocula | AO | BH | FF | GV | HL | HR | LP | RA |
| --- | --- | --- | --- | --- | --- | --- | --- | --- |
| AO | 0.07+0.02 | 0.06+0.04 | 0.06+0.05 | 0.03+0.02 | 0.11+0.05 | 0.03+0.02 | 0.03+0.02 | 0.02+0.02 |
| BH | 0.06+0.04 | 0.06+0.02 | 0.09+0.04 | 0.07+0.03 | 0.10+0.05 | 0.05+0.03 | 0.03+0.02 | 0.05+0.02 |
| FF | 0.06+0.05 | 0.09+0.04 | 0.12+0.02 | 0.09+0.03 | 0.02+0.02 | 0.07+0.04 | 0.07+0.03 | 0.05+0.03 |
| GV | 0.03+0.02 | 0.07+0.03 | 0.09+0.03 | 0.10+0.03 | 0.05+0.04 | 0.04+0.04 | 0.17+0.05 | 0.12+0.04 |
| HL | 0.11+0.05 | 0.10+0.05 | 0.02+0.02 | 0.05+0.04 | 0.10+0.03 | 0.03+0.02 | 0.08+0.05 | 0.08+0.05 |
| HR | 0.03+0.02 | 0.05+0.03 | 0.07+0.04 | 0.04+0.04 | 0.03+0.02 | 0.19+0.02 | 0.09+0.04 | 0.08+0.04 |
| LP | 0.03+0.02 | 0.03+0.02 | 0.07+0.03 | 0.17+0.05 | 0.08+0.05 | 0.09+0.04 | 0.13+0.04 | 0.13+0.04 |
| RA | 0.02+0.02 | 0.05+0.02 | 0.05+0.03 | 0.12+0.04 | 0.08+0.05 | 0.08+0.04 | 0.13+0.04 | 0.16+0.04 |
|  |  |  |  |  |  |  |  |  |
| Mixture(df=7,72) | 0.78 | 0.28 | 0.72 | 1.37 | 0.65 | 1.93 | 1.61 | 1.60 |

**Table S2** Plant yellowness in each mixed inoculum (mean + SE). Each row/column combination indicates the average biomass for this inocula combination. For each column, the F value from a one-way ANOVA is also presented. The shaded part of the table is presented twice. Plant species abbreviations are described in the material and methods section.

**Fig.S1** The ratio of predicted and observed effects of mixtures on chrysanthemum biomass (a) and yellowness (b). Means are shown (+/- 1 SE). Predicted effects of mixed inocula are calculated as (effect of inoculum A + effect of inoculum B)/2. Figures show the average effects of all mixtures which contained the respective monospecific inoculum. “Average” means the average effect of all mixed inocula, which is the same as presented in Fig.1. * represents significant difference from one sample t-test.

**Fig.S2** Relationship between the difference among the effects of monospecific inocula on plant biomass (a) and yellowness (b), and the difference between the observed and predicted effects of their mixtures (excluding mixtures which containing HR and monospecific HR inocula). The difference of monoculture inocula is calculated as an absolute value |effect of inoculum A – effect of inoculum B|. The difference between observed and predicted effects of the mixtures is calculated as (observed value of mixture A+B – predicted value of mixture A+B). The fit (R^2^) and *P* value of both regressions are also presented.

**Fig. S1**


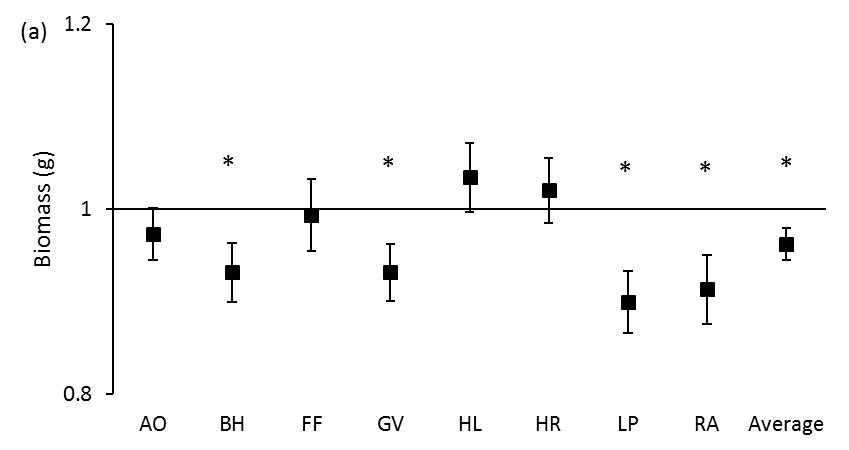

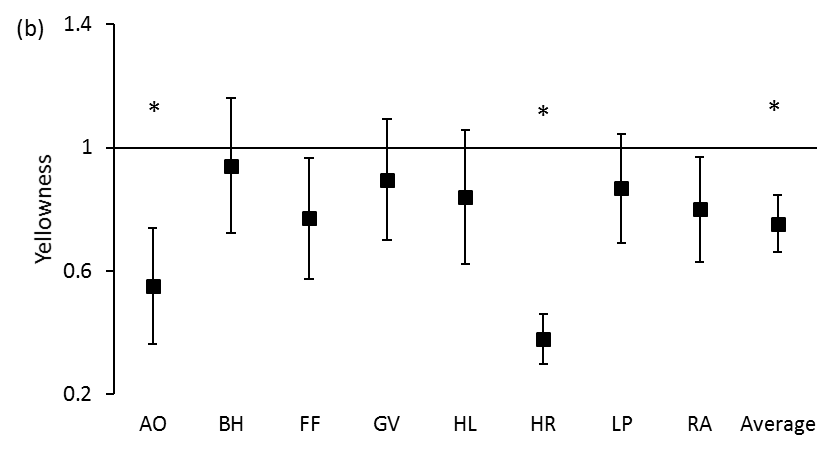


**Fig.S2**


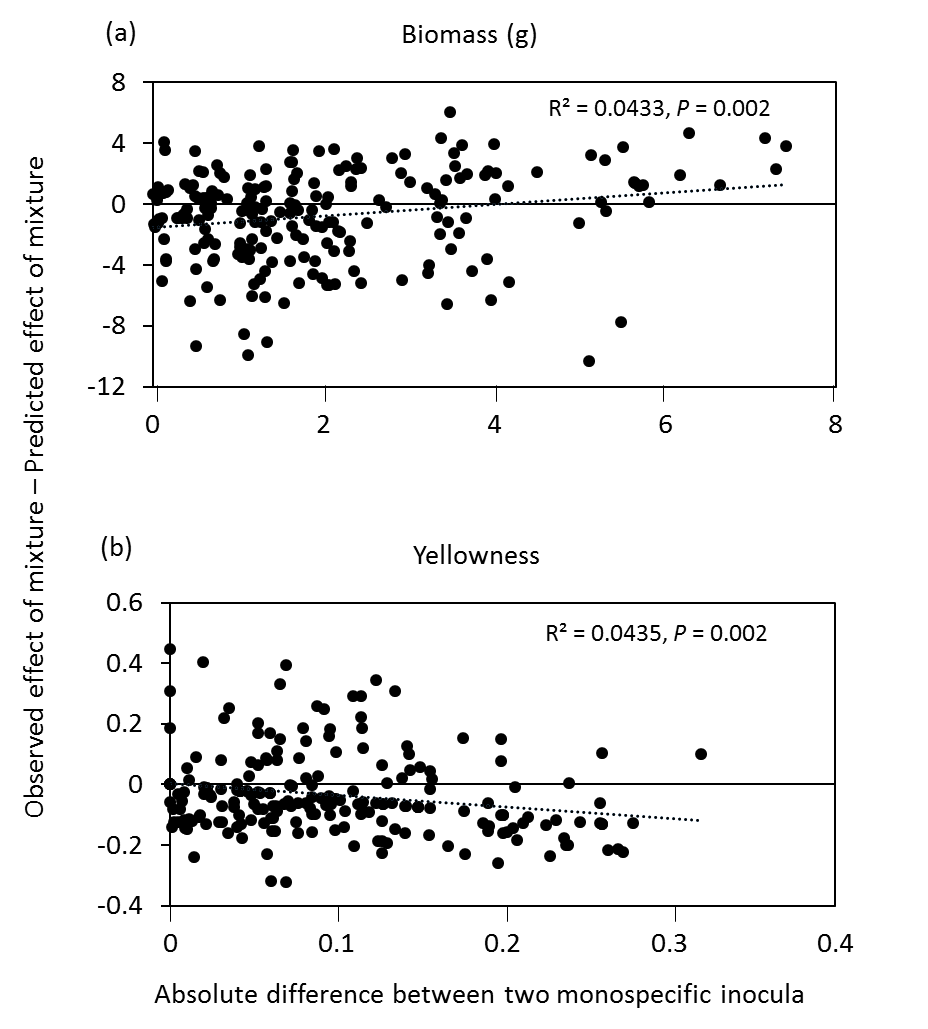

Supplement: Supplementary file 1 — (DOCX 115 kb) [file 11104_2018_3694_MOESM1_ESM.docx]
